# Peripheral sea-fan retinal neovascularization as a manifestation of chronic rhegmatogenous retinal detachment and surgical management

**DOI:** 10.1186/1471-2415-14-112

**Published:** 2014-09-23

**Authors:** Ilias Georgalas, Theodore Paraskevopoulos, Chyssanthos Symmeonidis, Petros Petrou, Chryssanthi Koutsandrea

**Affiliations:** Department of Ophthalmology “G.Gennimatas” Hospital, University of Athens, 59 Chrysanthemon str, 1545 Athens, Greece; Department of Ophthalmology, Aristotle University of Thessaloniki, Thessaloniki, Greece

**Keywords:** Chronic retinal detachment, Sea-fan neovascularization, Scleral buckling

## Abstract

**Background:**

To report the rare occurrence of peripheral retinal sea-fan neovascularization in a patient with chronic rhegmatogenous retinal detachment and describe the surgical management.

**Case presentation:**

A 29-year-old female patient was referred to our department by her ophthalmologist for investigation and treatment of peripheral retinal neovascularization in her right eye(RE). Visual acuity(VA) at presentation was 20/200 RE and 20/20 LE. Fundoscopy of the RE revealed a chronic inferotemporal retinal detachment and peripheral neovascularization with a sea fan configuration. Fundoscopy of the LE was without any findings. Fluorescein angiography confirmed the sea fan neovascularization in the RE with leakage of the newly formed vessels and peripheral ischemia while the LE did not demonstrate any neovascularization angiographically. Family history was negative for retinitis pigmentosa and haemoglobinopathies. Patient underwent full blood count and haemoglobin electrophoresis to exclude thrombocytosis and sickle cell anaemia, and serum angiotensin-converting enzyme (SACE) measurement to exclude sarcoidosis. Examination with scleral indentation of the RE revealed 2 peripheral small retinal holes close to the ora serrata . The patient underwent a scleral buckling procedure with a small segmental buckle limited to the area of the holes and cryotherapy. Ccryotherapy was not applied to the area with neovascularization and no subretinal fluid drainage was performed. The detached retina was successfully re-attached surgically and the subretinal fluid was gradually absorbed within three months from the time of surgery. Complete regression of neovascularization was evident 2 months postoperatively and VA improved to 20/30. Three years later the clinical and functional findings remain unchanged.

**Conclusion:**

Our case illustrates the rare but possible association of chronic retinal detachment with peripheral retinal sea-fan neovascularization; although the incidence is rare it may pose diagnostic and treatment dilemmas. In such cases and in the presence of retinal breaks, cryotherapy and a segmental buckle limited to the retinal holes and not on the neovascularization seems to suffice for the regression of the new vessels.

## Background

Rhegmatogenous retinal detachment is defined as the detachment between neurosensory retina and the pigment epithelium layer, caused by a retinal break or hole which allows fluid from the vitreous cavity to enter the subretinal space. Its incidence is estimated to be 1/10,000 people per year [1].

Although it is an acute, vision threatening condition, in rare cases it is presented in a chronic, slowly progressive form, which is not usually diagnosed until it has reached an advanced stage. Younger age and myopic refraction are factors related to the appearance of the chronic rhegmatogenous retinal detachment(RRD) [2, 3]. Peripheral retinal neovascularization is an extremely rare manifestation of chronic RRD [4, 5].

We report a case of a 29-year-old female myopic patient with a chronic rhegmatogenous retinal detachment, combined with sea-fan retinal neovascularization and describe its course after surgical management with scleral buckling.

## Case presentation

**Figure 1:**
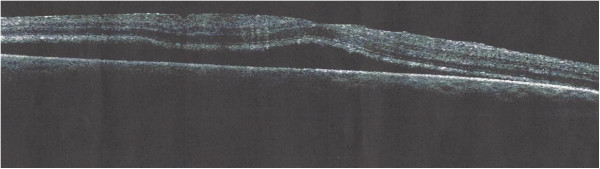
**Optical coherence tomography (OCT) of the posterior pole in the right eye demonstrating the presence of retinal detachment involving the macula.**

**Figure 2:**
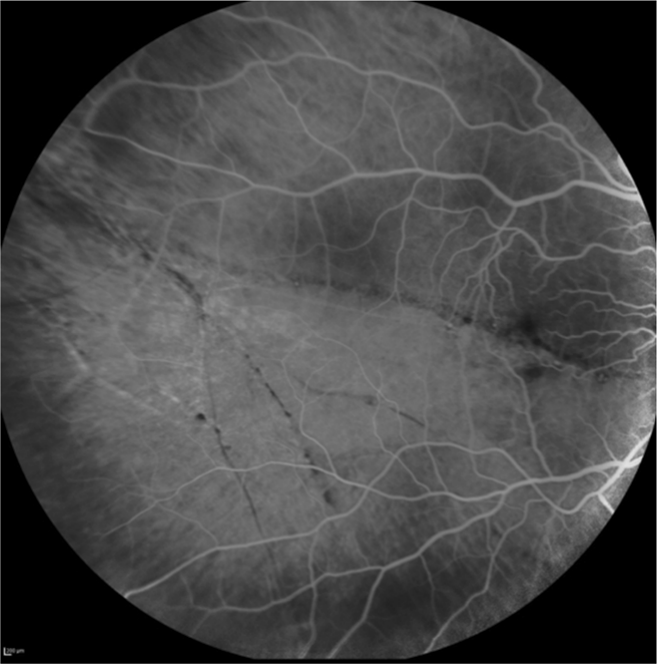
**Fluorescein angiography of the posterior pole of the right eye showing the chronic retinal detachment.**

**Figure 3:**
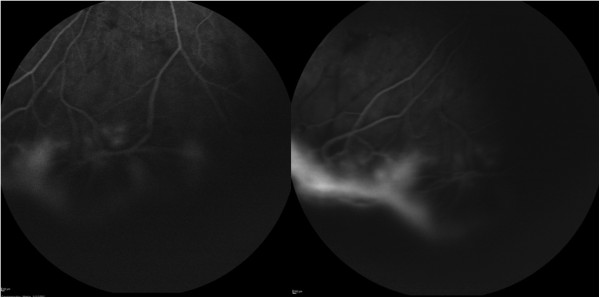
**Fluorescein angiography of the right eye demonstrating the neovascularization with sea-fan configuration (A) and leakage of the neovascularization (B).**

**Figure 4:**
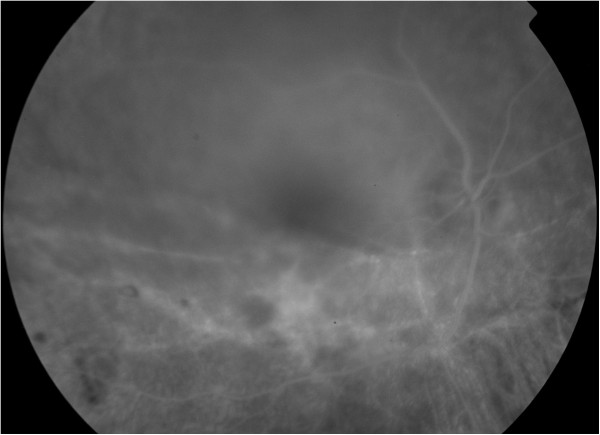
**Post-operative Fluorescein angiography of the right eye demonstrating regression of the neovascularization.**

A 29-year-old female patient was referred to our department for investigation and treatment of peripheral retinal neovascularization in her right eye(RE). She had been complaining of longstanding superior scotoma and a more recent decrease of RE visual acuity. She was a myope (spherical equivalent -9.25) with otherwise unremarkable ocular and general medical history. Her RE visual acuity at presentation was 20/200. Visual acuity of her left eye was 20/20. Fundoscopy of her RE revealed a chronic inferotemporal retinal detachment and significant neovascularization with sea-fan configuration. Left eye examination was unremarkable. Optical Coherence Tomography(OCT) (Figure 1) and Fluorescein angiography was performed and sea-fan neovascularization was confirmed on flourescein angiography, with leakage of the newly formed vessels and peripheral ischemia (Figures 2 and 3). The fellow eye did not demonstrate any signs of neovascularization. Family history was negative for retinitis pigmentosa and haemoglobinopathies. Patient underwent full blood count and haemoglobin electrophoresis to exclude thrombocytosis and sickle cell anaemia, and serum angiotensin-converting enzyme (SACE) measurement to exclude sarcoidosis. Examination with scleral indentation of the RE revealed 2 peripheral small retinal holes close to the ora serrata at 9 1/2 o’clock hours. After a discussion with the patient about risks and benefits of treatment, surgical management was undertaken. In the absence of posterior vitreous detachment, the patient underwent scleral buckling procedure with a small segmental buckle and cryotherapy limited to the area of the retinal holes; cryotherapy was not applied to the area with neovasularization and no subretinal fluid drainage was performed. The detached retina was successfully re-attached surgically and the subretinal fluid was gradually absorbed over a period of 3 months. Complete regression of neovascularization was evident 2 months postoperatively (Figure 4). Three years follow-up revealed a fully attached retina, patient’s visual acuity increased to 20/30 and sea fan neovascularization completely regressed.

## Conclusions

Chronic rhegmatogenous retinal detachment is a rare form of retinal detachment, with estimated prevalence estimated between 4, 5 – 29% [2]. This variation between several studies is attributed to various criteria selected in each study. Young age, myopic refraction, inferior position of detachment and the presence of small atrophic holes are considered as main risk factors for the appearance of chronic, asymptomatic rhegmatogenous retinal detachments [2, 3]. In rare cases they are combined with the presence of retinal microcysts and retinal neovascularization [4, 5].

Sea fan neovascularization is the hallmark of sickle cell retinopathy, but it is also reported in medical conditions, such as thrombocytosis [6], sarcoidosis [7], retinitis pigmentosa [8]. In the literature, this pattern of neovascularization has been very rarely associated with chronic rhegmatogenous retinal detachment; thus it should be differentiated from a non rhegmatogenous retinal detachment with secondary neovascularization (such as coats disease, peripheral retinoschisis, retinopathy of prematurity, familial exudative vitreoretinopathy) or a tractional retinal detachment associated with diseases secondary to Eales disease, or diabetic retinopathy [5]. Bonet in 1987 presented 9 eyes with peripheral neovascularization attributed to chronic retinal detachment. In all described cases the “neovascular formations were located just posterior to the retinal holes and in the close vicinity of the retinal holes” in contrast with our case where the neovascularization was located in the inferior detached retina and not close to the retinal holes. In the aforementioned study cryotherapy limited to the breaks was performed in 5 eyes when in the rest 4 eyes the area treated with cryotherapy included the neovascularization and/or the peripheral avascular retina and the scleral buckling extended to include the area of the neovascularization in all eyes [5].

Angiogenic factors such as vascular endothelial growth factor(VEGF) and basic fibroblast growth factor(bFGF), have been demonstrated to trigger the development of sea fan neovascularization in cases of sickle cell retinopathy [9]. In our case we postulate that hypoxia of the longstanding detached retina may have triggered the formation of new vessels, through increase of angiogenic factors, leading to a sea fan pattern of neovascularization.

Our case illustrates the rare but possible association of chronic retinal detachment with peripheral retinal seafan neovascularization which maybe located quite away from the retinal holes; although the incidence is rare it may pose diagnostic and treatment dilemmas. In such cases, cryotherapy and a segmental buckling limited to the retinal holes and not on the neovascularization seems to suffice for the regression of the new vessels.

## Consent

Written informed consent was obtained from the patient for publication of this case report and any accompanying images. A copy of the written consent is available for review by the Editor-in-Chief of this journal.

## References

[1] Fraser S, Steel D (2010). Retinal detachment. Clin Evid (Online).

[2] Li YM, Fang W, Jin XH, Li JK, Zhai J, Feng LG (2012). Risk factors related to chronic rhegmatogenous retinal detachment. Int J Ophthalmol.

[3] Orucov F, Galbinur T, Frenkel S, Landau D, Solomon A, Hemo I, Frucht-Pery J, Chowers I (2008). Prevalence of clinical asymptomatic retinal detachment in myopic population. Br J Ophthalmol.

[4] Labriola LT, Brant AM, Eller AW (2009). Chronic retinal detachment with secondary retinal macrocyst and peripheral neovascularization. Semin Ophthalmol.

[5] Bonnet M (1987). Peripheral neovascularization complicating rhegmatogenous retinal detachments of long duration. Graefes Arch Clin Exp Ophthalmol.

[6] Nobacht S, Cruysberg JR, Deutman AF (1999). Peripheral retinal nonperfusion associated with essential thrombocytosis. Am J Ophthalmol.

[7] Asdourian GK, Goldberg MF, Busse BJ (1975). Peripheral retinal neovascularization in sarcoidosis. Arch Ophthalmol.

[8] Kadayifçilar S, Eldem B, Kiratli H (2000). Retinitis pigmentosa associated with peripheral sea fan neovascularization. Acta Ophthalmol Scand.

[9] Cao J, Mathews MK, McLeod DS, Merges C, Hjelmeland LM, Lutty GA (1999). Angiogenic factors in human proliferative sickle cell retinopathy. Br J Ophthalmol.

[CR10] The pre-publication history for this paper can be accessed here: http://www.biomedcentral.com/1471-2415/14/112/prepub

